# Online Health Information Regarding Male Infertility: An Evaluation of Readability, Suitability, and Quality

**DOI:** 10.2196/ijmr.6440

**Published:** 2016-10-21

**Authors:** Stephanie Robins, Helena J Barr, Rachel Idelson, Sylvie Lambert, Phyllis Zelkowitz

**Affiliations:** ^1^ Jewish General Hospital Department of Psychiatry Montreal, QC Canada; ^2^ Ingram School of Nursing McGill University Montreal, QC Canada; ^3^ St.Mary's Research Centre Montreal, QC Canada; ^4^ Lady Davis Institute for Medical Research Montreal, QC Canada; ^5^ Department of Psychiatry McGill University Montreal, QC Canada

**Keywords:** male infertility, fertility preservation, Internet, online health information, men’s health

## Abstract

**Background:**

Many men lack knowledge about male infertility, and this may have consequences for their reproductive and general health. Men may prefer to seek health information online, but these sources of information vary in quality.

**Objective:**

The objective of this study is to determine if online sources of information regarding male infertility are readable, suitable, and of appropriate quality for Internet users in the general population.

**Methods:**

This study used a cross-sectional design to evaluate online sources resulting from search engine queries. The following categories of websites were considered: (1) Canadian fertility clinics, (2) North American organizations related to fertility, and (3) the first 20 results of Google searches using the terms “male infertility” and “male fertility preservation” set to the search locations worldwide, English Canada, and French Canada. Websites that met inclusion criteria (N=85) were assessed using readability indices, the Suitability Assessment of Materials (SAM), and the DISCERN tool. The associations between website affiliation (government, university/medical, non-profit organization, commercial/corporate, private practice) and Google placement to readability, suitability, and quality were also examined.

**Results:**

None of the sampled websites met recommended levels of readability. Across all websites, the mean SAM score for suitability was 45.37% (SD 11.21), or “adequate”, while the DISCERN mean score for quality was 43.19 (SD 10.46) or “fair”. Websites that placed higher in Google obtained a higher overall score for quality with an *r* (58) value of -.328 and a *P* value of .012, but this position was not related to readability or suitability. In addition, 20% of fertility clinic websites did not include fertility information for men.

**Conclusions:**

There is a lack of high quality online sources of information on male fertility. Many websites target their information to women, or fail to meet established readability criteria for the general population. Since men may prefer to seek health information online, it is important that health care professionals develop high quality sources of information on male fertility for the general population.

## Introduction

Infertility, defined as the inability to achieve a pregnancy after 12 months of unprotected sexual intercourse, is a public health problem affecting as many as 186 million people worldwide [1]. In approximately half the cases of infertility, male factors are the primary or contributing causes [2]. Nonetheless, many men lack basic knowledge about the causes of male infertility and the risks that infertility may pose to their own health [3]. This is also true for men who have undergone cancer treatment, which can have a devastating effect on sperm production. Men who are survivors of cancer are half as likely to father a pregnancy as men without cancer [4], yet many cancer patients are not well informed about the impact of their treatment and options for fertility preservation [5]. Male infertility has been associated with poorer general health, including obesity, diabetes, cardiovascular disease, as well as prostate and testicular cancer [6]. A diagnosis of infertility has a negative effect on men’s psychological and emotional well-being, contributing to elevated levels of sadness, stress, and anxiety, as well as distress in the couple relationship [7-10]. Male infertility is highly stigmatized, as it can be perceived as a threat to masculinity and may compromise men’s sense of identity [11]. This stigma may contribute to men’s reluctance to seek out information and support [12]. Instead, individuals with stigmatized illnesses are more likely to use the Internet to seek out health-related information [13]. Online searches for health information are particularly well-suited to meet the needs of men, who may prefer to obtain information anonymously and independently [14].

Internet use around the world continues to grow, with recent reports indicating 3.2 billion users worldwide, compared to 400 million users 15 years ago [15]. The growing body of online medical information has facilitated the search for health-related information independent from health care providers. A recent report indicates that up to 72% of Web users have looked up health information online [16]. However, for patients seeking fertility-related information, the quality is variable; online information rarely meets standards of accuracy, credibility, and navigability [17,18]. Moreover, many websites are often largely designed as advertisements, lack references [19], and have reading levels most appropriate for individuals with at least a high school education [20].

Standardized measures of readability, suitability, and quality have been developed to assess whether written information is appropriate for the general public. Readability refers to the level of ease with which a group of sentences can be understood by the reader [21]. It is recommended that material be written at a 6th to 8th grade level in order to be easily understood [22]. Suitability refers to whether written material facilitates comprehension for a given population, based on its content, literacy demand, graphics, layout and typography, learning stimulation/motivation, and cultural appropriateness [23]. The quality of health-related written material as a source of information on treatment options can be assessed by evaluating its clarity, reliability, and relevance [24].

Given the prevalence of male infertility, its association to physical and psychological well-being, and the likelihood that men may prefer to seek information about fertility online, it is important to evaluate online fertility and fertility preservation information. There is a paucity of research using standardized methods to evaluate websites related to male fertility. To our knowledge, no studies have used standardized tools to assess the readability, suitability, and quality of Canadian websites relating to male fertility and fertility preservation.

The present investigation examined (1) whether online information regarding male fertility and fertility preservation is generally readable, suitable, and of high quality; and (2) whether other factors such as geographic search location, website affiliation, audiovisual aids, advertising, and search engine result position are related to the website’s quality.

## Methods

### Sample

Given the very large number of websites that might be generated by a broad Internet search, in the interest of feasibility we sought to replicate how men might search for information. As a result, our online search strategy focused on (1) Canadian fertility clinic Websites (N=51); (2) major North American organizations related to fertility and fertility preservation (N=25); and (3) websites resulting from a Google keyword search (N=120). Our goal was to evaluate Canadian resources, and compare them to a sample of English language websites from countries other than Canada.

We opted to include the websites of all Canadian fertility clinics since these were most likely to be accessed and utilized by people seeking or engaged in treatment for infertility. Our team contacted fertility clinics and oncology centers across Canada to obtain a list of recommended websites where health care professionals refer patients for information on fertility and fertility preservation (personal communication P Zelkowitz). Our original sample size included 25 North American organizations such as the Canadian Fertility and Andrology Society, the Canadian Cancer Society, the Infertility Awareness Association of Canada, the American Society for Reproductive Medicine, and Resolve: the National Infertility Association.

**Figure 1:**
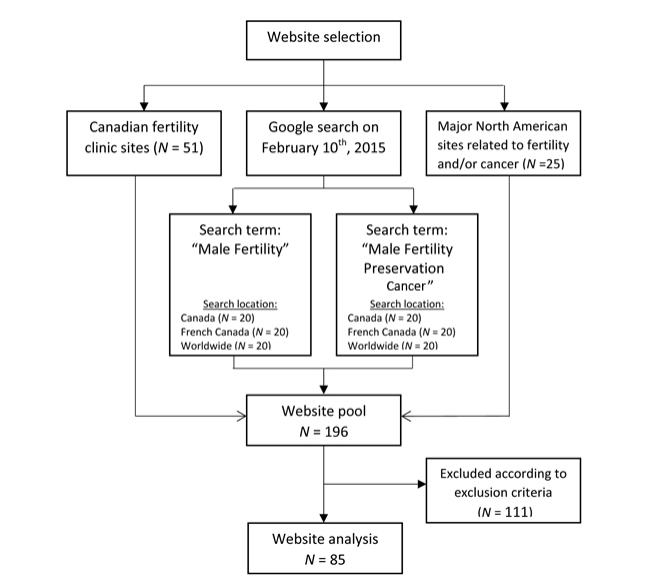
Website selection.

For comparison purposes, a cross-sectional online search was conducted on February 10th, 2015 from Montreal, Quebec using Google, which currently accounts for a 64.0% share of the explicit core search market [25]. Three separate location filters were applied: worldwide, English Canada, and French Canada. In order to ensure that the retrieval of Uniform Resource Locators (URLs) was not affected by our search history and existing preferences, all browsing history, cookies, and cache files were deleted before the query was undertaken. In consultation with a research librarian, we selected search terms that included the use of 1 noun [26] and a 2 to 4 total word count in English [27], with equivalent French terms for French language sites. We chose “male infertility” and “male fertility preservation cancer” for English sites and “infertilité masculine” and “préservation de la fertilité cancer hommes” for French language sites. We included the first 20 URLs for each location and search term, as per other website content analyses [28,29]. Our 3-pronged search strategy yielded a total of 196 websites, and excluded them if (1) the page was a magazine or journal article (N=28); (2) the site was not for a lay-searcher’s informational purpose (eg, a clinic website that only listed its opening hours; N=38); (3) the site only had information relevant to female fertility (N=15); (4) the site was a duplicate of a previously included website (N=24); or (5) it was a dead link (N=6). These criteria excluded 111 of the original pool of sites, leaving a final sample of 85 websites: 26 from the “male infertility” search, 18 from the “male fertility preservation” search, 28 Canadian fertility clinic websites, and 13 major North American organization websites (Figure 1). Website analysis was carried out between February and May 2015.

### Measures

#### Readability Indices

Readability scores were obtained using online readability analysis tools. The text from the entire website was copied into word processing software using the “text only” option, meaning features such as bold, italics, or hyperlinks were removed. All pictures, tables, and bullet points were also discarded, and lines of text following bullet points were not included if they did not form full sentences. This text was then pasted in its entirety into the automated readability tool. The online utility tool [30] used for English text analysis was extensively used to measure English readability in several Anglophone countries [21,31-33] and provided scores for each of the following reliable and validated readability formulae: the Fog Index, the Simplified Measure of Gobbledygook (SMOG), and the Flesch Kincaid Grade level [34-36]. These formulae calculate the grade level necessary to understand content based on the characteristics of text such as number of syllables per word and number of words per sentence; higher scores reflect lower readability. Average grade level readability scores were computed from the Fog, SMOG, and Flesch Kincaid Grade Level scores. The practice of averaging readability scores from several tools is widely endorsed [21,37,38] as a method of ensuring reliability.

French text was evaluated using the Système d'Analyse de Textes par Ordinateur (SATO) [39]. This tool was developed by researchers and government agents to assess French readability [40], and provides an adapted version of the Fog Index for the French language. This was the only automated readability score calculated for French content, given a lack of freely accessible online tools to calculate French text readability.

#### Suitability and Quality Ratings

Two trained raters completed suitability and quality assessments using the Suitability Assessment of Materials (SAM) and the DISCERN. These tools provide a standardized method for evaluating both print and electronic resources and have established reliability and validity.

The SAM checklist [23] is a 22-item measure that assesses how suitable material is for patient purposes based on 6 factors that affect both readability and comprehension. Content areas are assessed for how easily a reader understands the stated purpose of the material, the scope of information provided in relation to its purpose and the provision of examples of behavior that help problem-solve. This section also evaluates if an overall summary that retells key messages is provided, ideally using visual aids or examples. Section 2, literacy demand, determines the reading grade level determined using the Fry formula [41] and analyzes the complexity of the writing style reflected in sentence length, the “voice” of the narrative and its vocabulary. Ideal terms are common and explicit, offer non-value judgments, and bring forth mental images. The order of information is considered: context should be provided before new information is described and followed by an “advanced organizer” such as a header that informs the reader what to expect next. The third section, graphics, reviews all illustrations, charts, lists, and tables. Cover graphics are rated on their friendliness, attractiveness, and clarity of purpose, while illustrations are evaluated for complexity and if they provide familiar and easy to remember concepts. The relevance of illustrations is based on whether key messages are expressed in visual terms and without distraction. Other non-text information such as charts or tables are scored on whether or not they are coupled to a set of instructions for navigation and comprehension, and explained in a caption. Layout and typography, section 4, contains explicit criteria to determine if layout meets superior standards based in part on whether text and images are arranged in a logical sequence and are of an appropriate color, how contrast and white space is used, and if visual cuing (arrows, shading, boxes) is incorporated. Type font and size, along with whether or not information is divided into manageable subsections, is evaluated. Section 5, learning stimulation and motivation, assesses whether readers are asked to actively engage with the material by asking questions or by problem-solving, whether appropriate behavior or skills are clearly modeled, and how well topics are divided into understandable or doable components that might ultimately lead to reader self-efficacy. The final section of the SAM determines the cultural appropriateness of the material by assessing how well the logic, language, and experience of the instruction and the reader match. In addition, images or examples of this culture should be presented in a positive light, not exaggerated nor caricatured.

In all categories, items are rated from 0 to 2, where higher scores reflect increased suitability. To obtain a total score, individually scored items are summed and divided by the total possible score for those items, which is represented as a percentage. These total scores are interpreted as follows: ≥70% “superior”, 40% to 69% “adequate”, and <40% “not suitable”. This tool has been extensively used in the assessment of Web-based health education materials by researchers and government bodies [42,43].

Researchers modify the SAM for on-screen patient educational material assessment by excluding individual items (as outlined in the original Doak and Doak scoring manual) while others [43] have omitted entire categories that are not applicable. One study replaced typography features such as the contrast of paper to typeface with online links [44]; we did not modify the original scoring rubric for the analyses performed here and omitted items that were not applicable on a site by site basis.

The 16-item DISCERN instrument [24] assesses the quality of material as a source of information on treatment choices. The DISCERN is divided into 3 subsections. Section 1 (questions 1-8) addresses the reliability of the source and investigates whether the document achieves a clearly articulated aim, the information is relevant and provides appropriate options to patients, the material is evidence-based, dated, and has been externally reviewed or is from a variety of sources and is unbiased, the information covers areas of uncertainty and finally, other relevant resources are provided. Section 2 (questions 9-15) assesses treatment options and describes how the treatment acts on the condition and may affect the patient, the benefits and risks of treatment, how a condition might progress without medical management, the quality of life that can be expected depending on treatment choice, if it is clear that different options exist, and finally, how to proceed with shared decision making which may include family, friends, or other health care professionals. Section 3 is composed of one final question that provides an overall rating for the material. Each item is rated from 1 to 5, where higher scores reflect higher quality. The DISCERN tool does not specify how to present total scores, but a majority of researchers have presented sum scores of the first 15 items (ranging from 15 to 75), and categorized total scores as “excellent” (63-75), “good” (51-62), “fair” (39-50), “poor” (27-38), and “very poor” (15-26) [45-48]. The DISCERN tool has demonstrated high internal consistency (Cronbach alpha = .78) and interrater agreement in total scores (ICC = .82, *P*<.001), as well as convergent validity by being significantly related to total scores using a different measure of material quality (*r*=.53, *P*<.001) [49].

In the present study, interrater reliability (IRR) was calculated for both the SAM and the DISCERN based on a sample of 15 websites coded by both raters (17.6% of the total sample). IRR was assessed for mean subsection scores for both scales using two-way single-measures mixed effects intraclass correlation (ICC) for absolute agreement, where higher values reflect higher IRR. According to the interpretation guidelines summarized by Cicchetti [50], our ICC scores ranged from fair (ICC=.533) to excellent (ICC= .828). Disagreements in scores were resolved by consensus ratings. A summary of IRR statistics can be found in Table 1.

**Table 1:** Summary of interrater reliability scores for the Suitability Assessment of Materials and DISCERN assessments (N=15).

Scale		ICC^a^	*P* value
**DISCERN**			
	Section 1-Reliability (items 1-8)	.828	.000
	Section 2-Treatment choices (items 9-15)	.581	.009
	Section 3-Overall rating (item 16)	.812	.000
**SAM^b^**			
	Section 1-Content	.533	.019
	Section 2-Literacy demand	.817	.000
	Section 3-Graphics	.752	.001
	Section 4-Layout and typography	.605	.008
	Section 5-Learning stimulation, motivation	.636	.001
	Section 6-Cultural appropriateness	.715	.001

^a^ICC: intraclass correlation

^b^SAM: Suitability Assessment of Materials.

#### Other Variables

Other characteristics that were assessed included website affiliation (government, university/medical, non-profit organization, commercial or corporate, private practice), position in Google, year last updated, and whether or not sites contained information for men. We noted if sites contained advertising: announcements such as banner images, animations, text, or images that redirected to outside websites and which were designed to sell a commodity or service. The presence of audiovisual aids such as embedded videos or animation, podcasts, or audio recordings was also evaluated.

Data were analyzed using the Statistical Package for the Social Sciences (IBM SPSS Statistics for Windows version 19.0, Armonk, NY). Critical value of significance was determined at *P=*.05.

## Results

### Descriptive Statistics

A summary of descriptive statistics can be found in Table 2. Websites were compared according to “website category” (Canadian fertility clinics, major North American websites and Google keyword search) and “website location” (worldwide, English Canada, and French Canada).

Chi-square analysis demonstrated that there were significant differences among website categories on the exclusion criteria (X^2^_15_=59.53, *P*<.001, N=98). Further analyses indicated that Canadian fertility clinic websites were significantly more likely than non-clinic websites to be excluded because they only contained information on female fertility, (X^2^_1_=15.65, *P*<.001, N=98); 20% (10/51) of clinic websites met this exclusion criterion, compared to 2.5 % (3/120) of the sites found through Google searches, and 8% (2/25) of major North American organizations.

#### Readability

The mean readability score for the entire sample was grade level 14.19, meaning that the content was geared to readers with at least some university education. The French language readability tool (SATO) calculated 16.62 years of education were necessary for French sites to be easily understood, equivalent to the “proficient user” level (C1-C2) as defined by the Common European Framework of Reference for Languages [51]. Overall, readability scores ranged from grade level 10.19 to 20.40, demonstrating that none of the websites were appropriate for the recommended 8th grade level readability.

#### Suitability and Quality

Only 1 (1%, 1/85) website met the criteria for “superior” suitability as established by the SAM, 61 (72%, 61/85) met the criteria for “adequate” suitability, and 23 (27%, 23/85) were deemed “not suitable”. The sample-wide mean SAM score was 45.37% (SD 11.21), indicating “adequate” suitability.

Descriptive statistics for individual items on the SAM can be found in Table 3. The highest suitability scores were for items related to the placement of context (2d), visual navigation aids (2e) typography (4c), and motivation (5c). Items that rated low in suitability included content about behavior (1b), summaries (1d), reading grade level (2a), the relevance of illustrations (3c), and cultural images (6b). Overall, many sites failed to provide illustrations or other graphics, as revealed by missing scores in section 3.

For the DISCERN instrument, only 3 websites met the criteria for “excellent” quality, 21 were “good”, 32 “fair”, 25 “poor”, and 4 “very poor”. The sample-wide mean DISCERN score was 43.19 (SD 10.46), which falls in the lower end of the “fair” range. Mean scores of DISCERN items are found in Table 4.

**Table 2:** Summary of descriptive and frequency statistics for the final sample of websites.

Variable		N^a^	Mean (SD^b^)	Frequency (%)
Website location		85		
	Canada			38 (45)
	French Canada			35 (41)
	Worldwide			12 (14)
Website category		85		
	Canadian fertility clinics			28 (52)
	Major North American			13 (15)
	Keyword search on Google			44 (59)
Website affiliation		85		
	Government			4 (5)
	University or medical			19 (22)
	Non-profit organization			19 (22)
	Commercial or corporate			7 (8)
	Private practice			36 (42)
Presence of ads		85		10 (12)
Presence of audiovisual material		85		9 (11)
Fry		73	15.09 (1.57)	
Fog		73	14.59 (2.04)	
SATO^c^		12	16.62 (2.00)	
SMOG^d^		73	13.92 (1.45)	
Flesch-Kincaid grade level		73	12.89 (1.76)	
Average grade level		84	14.23 (2.04)	
SAM^e^ percentage score		85	45.37 (11.21)	
DISCERN total 1-15		85	43.19 (10.46)	
DISCERN question 16		85	2.61 (1.11)	

^a^N: sample size.

^b^SD: standard deviation.

^c^SATO: Système d'Analyse de Textes par Ordinateur.

^d^SMOG: Simplified Measure of Gobbledygook.

^e^SAM: Suitability Assessment of Materials.

**Table 3:** Descriptive statistics of the Suitability Assessment of Materials items.

SAM^a^ factor		N^b^ (%)			
		0^c^	1^c^	2^c^	Missing
**Content**					
	1a) Purpose is evident	3 (3)	62 (73)	20 (24)	
	1b) Content about behaviors	65 (77)	20 (23)		
	1c) Scope is limited	20 (24)	29 (34)	36 (42)	
	1d) Summary or review included	78 (92)	1 (1)	6 (7)	
**Literacy demand**					
	2a) Reading grade level	85 (100)			
	2b) Writing style	44 (52)	33 (39)	8 (9)	
	2c) Common vocabulary	43 (51)	33 (39)	9 (10)	
	2d) Context given first	4 (5)	22 (26)	59 (69)	
	2e) Use of “road signs”	5 (6)	10 (12)	70 (82)	
**Graphics**					
	3a) Cover graphic shows purpose	10 (12)	30 (35)	8 (9)	37 (44)
	3b) Type of graphics	14 (16)	16 (19)	4 (5)	51 (60)
	3c) Relevance of illustrations	72 (85)	11 (13)	2 (2)	
	3d) Lists, tables, etc explained	4 (5)	6 (7)	3 (3)	72 (85)
	3e) Captions used for graphics	11 (13)	8 (9)	5 (6)	61 (72)
**Layout and typography**					
	4a) Layout factors	22 (26)	50 (59)	13 (15)	
	4b) Typography	1 (1)	8 (9)	76 (90)	
	4c) Subheadings used	32 (38)	24 (28)	29 (34)	
**Learning stimulation, motivation**					
	5a) Interaction used	42 (49)	43 (51)		
	5b) Behaviors modeled/specific	43 (51)	30 (35)	12 (14)	
	5c) Motivation and self-efficacy	7 (8)	15 (18)	63 (74)	
**Cultural appropriateness**					
	6a) Match in logic, language, experience	16 (19)	44 (52)	25 (29)	
	6b) Cultural images and examples	65 (76)	20 (24)		

^a^SAM: Suitability Assessment of Materials.

^b^N: sample size

^c^Scoring: 0= not suitable, 1=adequate, 2=superior.

**Table 4:** Descriptive statistics of the DISCERN items.

Item		Mean (SD^a^)
**Reliability**	
	1) Aims clear	2.67 (1.02)
	2) Aims achieved	3.14 (0.74)
	3) Relevance	3.91 (1.03)
	4) Sources of information clear	2.05 (1.14)
	5) Clear when information was produced	2.29 (1.74)
	6) Balanced and unbiased	2.96 (0.76)
	7) Details of additional sources	3.84 (1.74)
	8) Refers to areas of uncertainty	2.74 (1.66)
**Treatment choices**	
	9) Describes how treatments work	3.64 (1.24)
	10) Describes benefits of treatments	2.93 (1.35)
	11) Describes risks of treatments	2.16 (1.43)
	12) Describes what would happen without treatment	1.59 (1.20)
	13) Describes how treatments affect quality of life	2.48 (1.48)
	14) Clear that there may be more than one treatment choice	3.80 (1.36)
	15) Supports shared decision-making	2.99 (1.48)
**Overall rating**	
	16) Overall quality	2.61 (1.11)
Range, 1-5	

^a^SD: standard deviation

**Table 5:** Summary of correlations between website assessment measures.

Variable	1	2	3	4
1) Mean grade level readability	1.00			
2) SAM^a^ total score	-.389^b^	1.00		
3) DISCERN total score	-.280^c^	.484^b^	1.00	
4) DISCERN question 16	-.308^d^	.545^b^	.852^b^	1.00

^a^SAM: Suitability Assessment of Materials.

^b^*P<*.001.

^c^*P=*.010.

^d^*P=*.004.

Items that rated highly in terms of quality included the material being suited to user needs and thus relevant (3), the provision of further sources of support or information (7), a description of how treatment acts on the condition and may affect the patient (9), and that there may be more than one choice of treatment (14). Items that scored lowest in terms of fulfilling the criterion were clarity of sources (4), description of risks of treatment (11), and what would happen without treatment (12).

#### Year Last Updated

Only 28 websites (33%, 28/85) reported the year of the last update.

### Readability, Suitability, and Quality

#### Intercorrelations

Associations between different measures of readability were tested using Pearson correlation coefficient. The mean grade level readability score, SAM score, DISCERN total score, and DISCERN question 16 score were all significantly correlated. A summary of correlations can be found in Table 5. This demonstrates some convergent validity for the measures used to assess the websites.

#### Relationships Between Location or Search Category

Using one-way analysis of variance, we examined whether classification of website location (French Canada, English Canada or worldwide) or category (Canadian fertility clinic, major North American site, Google keyword search), were related to differences in our measures of content.

A significant between-group difference was found for average readability based on location, (*F*_2,82_ = 12.87, *P<*.001). Post-hoc analyses using Tukey’s honest significant difference (HSD) demonstrated that the location “French Canada” had a significantly (*P<*.001) higher mean readability score of 16.66 (SD 2.14) than both “Canada” and “worldwide” locations with mean values of 13.91 (SD 1.60) and 13.73 (SD 1.88), respectively, indicating that French Canadian websites were less readable. Neither suitability nor quality ratings differed by search location.

Mean readability differed significantly across search categories (*F*_2,70_ = 3.27, *P=*.05). In Tukey’s HSD post-hoc analyses, major North American websites had a mean grade reading level of 12.91 (SD 1.84), significantly lower than Canadian fertility clinics with a mean value of 14.32 (SD 1.36, *P*=.04). However, they were not different from Google keyword search sites (*P*=.28) with a mean value of 13.76 (SD 1.86).

There was also a marked difference in quality for sites from different categories (*F*_2,82_ = 5.46, *P=*.006). Major North American sites had a significantly higher DISCERN mean score of 51.23 (SD 8.88) than clinic sites with a mean score of 40.36 (SD 9.30, *P=*.002) or Google keyword search websites with a mean score of 42.61(SD 10.59, *P=*.007). Search category was not related to suitability.

#### Website Affiliation

Sites were grouped based on their association with a private practice, government, a corporation or commercial enterprise, a university or medical society, or a non-profit organization. Readability, quality, and suitability did not differ based on affiliation.

#### Google Keyword Search

The first 20 sites retrieved by Google were scored in the order they appeared, with the highest rank receiving the score of 1, or first. Google positioning was not associated to measures of readability or suitability with *r* (58) values of -.144 (*P*=.282) and -.151 (*P*=.257), respectively; however, higher positioning in Google was significantly correlated to the overall DISCERN score with an *r* (58) value of -.328 (*P=*.012).

#### Advertisements, Audiovisual Aids

We also investigated whether overall readability, SAM, and DISCERN scores differed according to presence of advertisements, or presence of audiovisual material. Sites that contained advertisements were not different in readability (*t*_10_=0.798, *P*=.449), suitability (*t*_10_=-0.021, *P*=.984) nor overall quality rating (t_83_=1.56, *P*=.122), than those without. Websites that incorporated audiovisual aids were also not significantly different from those that did not in relation to readability (*t*_83_=0.248, *P*=.805), suitability (*t*_83_ =0.152, *P*=.880), and quality (*t*_83_=-0.426, *P*=.671) scores

## Discussion

### Principal Findings

This review examined the readability, suitability, and quality of Canadian and international websites relating to male infertility and male fertility preservation, using standardized measures. Overall, automated readability indices demonstrated that online information regarding male infertility and male fertility preservation had very low readability and was most appropriate for individuals with post-secondary levels of education. In terms of suitability, only one site rated as superior, while the majority (72%, 61/85) had overall scores of “adequate”. Only one third of websites reported the year of their last update, a scant 11% (9/85) of sites made use of audio visual material, and approximately 20% (10/51) of Canadian fertility clinic websites did not include information for men. Between search categories, the major North American organizations scored highest in quality ratings and had the easiest English language readability, followed by the Google search and Canadian clinics. Google ranking was found to be associated with quality but not readability nor suitability.

Online health care resources have transformed how the public manages their health as well as how they navigate patient-doctor interactions. Patients turn to the Internet prior to consulting health care providers in order to better understand their own condition [52], and bring information about possible diagnoses, tests, and treatments to health care professionals for interpretation and advice [53]. The Institute of Medicine established six core elements underlying patient-centered care in a 2001 report titled *Crossing the Quality Chasm: A New Health System for the 21st Century* [54]. Two of these elements, “information, communication, and education” and “respect for patients’ values, preferences, and expressed needs”, can be fulfilled by electronic health (eHealth) resources. For example, patients in Canada can access information on many fertility websites in both English and French, which may result in better comprehension and thus better decision making. In one pilot project, a European fertility clinic implemented an online fertility community alongside their practice and found that patients appreciated having online access to their test results as well as the ability to communicate with medical professionals for interpretation and planning [55]. However, online information must be available, evidence-based, and explained in a language or platform patients can interpret easily and correctly. Many of the websites we evaluated did not meet these criteria; quality ratings indicated that many websites do not specify the source of the information, or the risks associated with treatment as well as the risk to well-being without treatment.

According to The Canadian Public Health Association (CPHA) Expert Panel on Health Literacy, more than half (55%) of Canadian adults in 2008 were considered to have “less than adequate literacy skills” [56]. This same report demonstrated that low health literacy is associated with worse health outcomes. All content presented in the 85 sites we examined required reading comprehension ability well beyond that of the general public. There is thus an imperative to not only simplify text, but also move beyond reading level to make Web-based material accessible and user friendly by integrating multimedia strategies. Illustrations, when added to difficult text, improve patient recall and informed decision making [57]. We found medical terminology was not often accompanied by illustrations or relevant graphics as noted by the suboptimal scores and missing values of the SAM category “graphics”. Other items of suitability that may help to make content relevant and understandable, including summaries of key messages, behavior modeling, and cultural considerations were also inadequate.

Educational videos and animation employed in the context of health care have been shown to increase patients’ knowledge of their own condition and their compliance with care [58,59], recall of oncology information [60], decision making, and self-efficacy [61]. However, audio visual material was only incorporated into 11% (9/85) of the sites evaluated. Interactive components, spoken text, animation, or video may also help reach people of diverse cultural backgrounds and for whom English or French is not their first language.

While most fertility clinic websites provided information for men, 20% (10/51) did not, suggesting that websites maintained by these clinics may be more tailored to women. Reproductive health tends to be conceptualized as a “women’s issue”, particularly since fertility treatment is primarily focused on women’s bodies, even when male factors are implicated [62]. In a review of urology websites [63] the authors recommend that health care providers work towards ethically balanced and educational websites for their patients, suggesting that websites be built incorporating the standard such as the Health On the Net accreditation seal [64]. They also recommend that medical boards guide doctors in good practice by creating policy regarding online media; this kind of committee could address issues such as gender bias. While clinic websites presented the most complex language and had, on average, the lowest quality ratings, major North American organizations that had been recommended by medical professionals performed best in terms of readability and overall quality, with the highest ranking being the Mayo Clinic website, the Resolve website, and the Cancer(.org) website. It may be that these organizations have the necessary resources to invest in creating an effective online presence. Health care providers have an important role to play in recommending specific websites to their patients, who appreciate such guidance in navigating online information [65,66].

Web searchers appreciate educational material that is retrieved and rank-ordered quickly [67]; however, there may be an assumption by consumers that higher ranked sites are more relevant and trustworthy [68]. In the present analysis, positioning in the Google search results was not a reliable indicator of website readability or suitability but was associated with overall quality. Google determines how to retrieve and rank pages using sophisticated methods that are customized to the individual and that consider user location, preferences, and history. Their algorithm PageRank is a tool that provides a measure of site popularity by identifying pages that have been linked to by others and this link is weighted to the importance of that source [69]. This process has recently been investigated as a method to rank and navigate biomedical literature [70]. There is some evidence that higher ranked sites describing medical information have higher quality content and easier readability [71,72]; however, others do not corroborate these findings [73,74]. To our knowledge, few analyses of website content include search engine ranking, but this area should be investigated further as patients often equate popular sites with reliable information.

### Limitations

There were several limitations to the present study. The focus of the study was to assess the quality of Canadian online sources of information about male infertility and male fertility preservation, with a sample of international sources included for comparative purposes. A comprehensive examination of international websites was beyond the scope of this study. As a result, the results of this study may not be applicable to fertility clinic websites and other online resources in other countries. Replication of these analyses in other countries would be required. Furthermore, we measured readability, suitability, and quality of website content, but not the validity of this information. While our interest was whether information was appropriately presented for the general population, future research should seek to establish the accuracy of online male fertility information. Our study found that French Canadian websites were significantly less readable than English Canadian or international websites. This result may be due to the fact that a different measure was used to assess readability, as no other significant differences in suitability or quality was found between location groups. We also had only one tool at our disposal to assess the readability of French websites, and scores using this tool were consistently higher than English website Fog index values. It is unclear whether these differences were due to the tool, and future studies should seek to assess the validity of a readability calculator for the French language.

### Conclusions

In general, the findings of this study suggest that there is a lack of appropriate and optimal sources of fertility information for men. Our analysis took into consideration the multidimensional nature of health literacy requirements; we considered aspects of reading grade level, measures of suitability, quality, search engine rank, sponsorship, audiovisual aids, and whether content was relevant and up to date. Our results highlight specific aspects of websites that need improvement, thereby providing information that can inform the development of Web-based resources in this domain. These features of websites may contribute to how a patient determines the credibility of the information they retrieve when seeking evidence that guides decision making. As men with fertility health concerns take on the responsibility of making informed choices about self-care, the burden also lies with medical professionals, website developers, and health care agencies to develop high quality sources of information to guide these choices.

## Abbreviations

HSD: honest significance difference
ICC: intraclass correlation
IRR: interrater reliability
SAM: Suitability Assessment of Materials
SATO: Système d'Analyse de Textes par Ordinateur
SMOG: Simplified Measure of Gobbledygook
URL: Uniform Resource Locator
